# A Comparative Study of Exceptional Experiences of Clients Seeking Advice and of Subjects in an Ordinary Population

**DOI:** 10.3389/fpsyg.2013.00065

**Published:** 2013-02-18

**Authors:** W. Fach, H. Atmanspacher, K. Landolt, T. Wyss, W. Rössler

**Affiliations:** ^1^Institute for Frontier Areas of Psychology and Mental HealthFreiburg, Germany; ^2^Collegium HelveticumZurich, Switzerland; ^3^Psychiatric University Clinic ZurichZurich, Switzerland

**Keywords:** continuum hypothesis, exceptional experiences, mental disorders, phenomenological patterns

## Abstract

Exceptional experiences (EE) occur frequently within the populations of many countries and across various socio-cultural contexts. Although some EE show similarities with mental disorders, it would be a mistake to identify them in general as disorders. In fact, the vast number of individuals reporting EE includes subclinical and completely healthy subjects. We conducted a comparative empirical study of several characteristics of EE for two samples – one from ordinary population and the other from clients seeking advice. We found surprisingly similar phenomenological patterns of EE in both samples, but the frequency and intensity of EE for clients seeking advice significantly exceeded those for the ordinary population. Our results support the hypothesis of a continuous spectrum between mental health and mental disorder for the types of experiences analyzed.

## 1 Introduction

Exceptional (or extraordinary) experiences (EE) are usually understood as deviations from what might be referred to as ordinary experiences, i.e., experiences consistent with typical “reality models” (Metzinger, 2003) that individuals develop to cope with their socio-cultural environment. In modern societies, basic elements of such models are established epistemological concepts (such as cause-and-effect relations) and scientific principles and laws (such as gravitation). Experiences inconsistent with those basic elements are considered exceptional.

A well-known example of EE is the meaningful coincidence of events with no causal connection which Jung (1955) referred to as synchronistic. Another example, widely studied recently (cf. Metzinger, 2005) concerns out-of-body experiences, where an individual’s material body is typically “seen” by the same individual from outside his body. The entire spectrum of EE is more comprehensively introduced in Sec. 2.1.

Contrary to naive expectations, EE are not rare but abundant. Several studies (Greeley, 1975; Gallup and Newport, 1991; Haraldson and Houtkooper, 1991; McClenon, 1994; Newport and Strausberg, 2001; Schetsche and Schmied-Knittel, 2005; Belz, 2009) have estimated frequencies of 30–50% for populations in Western countries, and higher than that within other cultural contexts. It is unclear, however, how the willingness of individuals to reveal EE can be faithfully assessed.

EE as such, and their phenomenology in particular, must be systematically distinguished from the belief in experiences, such as magical ideation (Eckblad and Chapman, 1983). Although experiences and beliefs are correlated, individuals with strong beliefs in non-causal influences or unknown physical forces (Lindeman and Aarnio, 2006) do not necessarily report EE (and vice versa). Moreover, EE tend to be more or less temporary states whereas beliefs are usually regarded as persistent traits.

Numerous studies have shown that belief in EE is correlated with high scores on scales measuring dispositions for absorption (Tellegen and Atkinson, 1974), fantasy proneness (Wilson and Barber, 1983; Rao, 1992), field dependence and suggestibility (Hergovich, 2003), transliminality (Thalbourne, 2000), hypnotizability (Hilgard, 1974; Lynn and Sivec, 1992), imagination (Lynn and Rhue, 1986, 1987), anxiety (Wolfradt, 1997), or dissociation (Spiegel and Cardeña, 1991; Frischholz et al., 1992).

Individuals reporting EE and altered states of consciousness also show tendencies toward schizotypy (Pizzagalli et al., 2001), dissociation (Richards, 1991), transliminality (Sherwood and Milner, 2004–2005), hypersensibility (Thalbourne, 2000; Jawer, 2006), and “thin-boundariedness” (Hartmann, 1991). However, it is unknown what type of precise relationship a particular EE may have with particular beliefs and personality traits.

Both EE and magical beliefs may be associated with phases of mental disorder (Eckblad and Chapman, 1983; Thalbourne and Delin, 1994; Berenbaum et al., 2000). Nevertheless, even if there is a substantial overlap with many symptoms and diagnoses of mental disorders, EE *per se* must not be categorized as such. For instance, schizotypy is not necessarily connected with mental health problems, and hearing voices is not psychotic in general (Romme and Escher, 1989, 1996; Bentall, 1990, 2000). An overall survey of published studies has shown that the evidence for a relationship between mental disorders and EE is inconsistent and ambiguous (Belz and Fach, 2012).

It has recently been proposed (van Os et al., 2000; van Os, 2003) that a continuous spectrum – from sound mental health to mental disorder – reflects the situation more properly than a discrete distinction between them. Support for this continuum hypothesis derives from several studies, as reviewed by van Os et al. (2009). Accordingly, EE may or may not be symptoms of mental disorder; similarly, they may or may not even be indicators or precursors of such disorders. Although significant relationships can be found between EE, critical life events and traumatic experiences (Irwin, 1992, 1993, 1994; Perkins and Allen, 2006), or even between actual life conditions and specific patterns of EE (Belz and Fach, 2012), the conditions for such correlations are still unknown.

Here we present additional evidence, based on particular types of EE, for the continuum hypothesis. Different from van Os et al. (2009), who focused on severely and less severely disordered individuals, we are concerned with the “healthy” side of the spectrum. We examined the phenomenology, frequency, and intensity of EE within ordinary population as well as in subclinical (i.e., non-diagnosed) individuals seeking advice.

The following section 2 provides some theoretical and empirical background for a systematic classification of the phenomenology of EE. Section 3 presents the empirical basis for our study. Section 4 describes the resulting classes of EE and the contexts within which they occur. We compare their frequencies and intensities and discuss some conclusions and implications of our results in Section 5.

## 2 Background

### 2.1 Theoretical background

A recently proposed classification of EE (Fach, 2011; Belz and Fach, 2012) is based on a few key postulates of Metzinger’s (2003) theory of mental representations. These representations are elements of a *model of reality* that subjects create, develop, and modify during their lifetime. Two fundamental components of this model, or two major representations within it, are the *self model* and the *world model*. (Contrary to Descartes’ ontologically conceived dualism, Metzinger’s distinction is explicitly epistemic.)

The world model contains all representations that a subject has developed about states of the material world, including his or her own bodily features. As a matter of principle, the referents of these representations are also observationally accessible to other individuals. Thus, intersubjective knowledge (sometimes called “objective” “third-person” knowledge) about them is possible.

The self model comprises all representations that a subject has developed about his or her internal states, such as sensations, cognitions, volitions, affects, motivations, and inner images. Knowledge about these states is private and, as a rule, can be experienced only by that subject itself, because it is “subjective” and based on “first-person” accounts.

Although the world model and the self model are separate elements within the overall reality model, their referents are often experienced as correlated. For instance, a subject’s bodily organs or limbs (represented by a subject’s world model) and bodily sensations (represented by a subject’s self model) are usually experienced to be mutually related to one another.

Nevertheless, a subject can distinguish self and world. Mental states induced by external sensory stimuli differ from states generated by internal processing. This is why, for example, touching a hot stove (represented in the world model) can be distinguished from the experienced pain (represented in the self model). In this sense (ordinary) subjects are capable of differentiating their inner images, affects, and fantasies from their perception of material events in the external world.

EE typically appear as deviations^1^ in a subject’s reality model. This entails a classification of EE based on two pairs of phenomena (cf. Fach, 2011; Belz and Fach, 2012). One pair refers to deviating experiences within the subject’s self model and world model, while the other refers to the way in which elements of those models are merged or separated above or below ordinary (“baseline”) correlations. The resulting four classes of EE can be characterized as follows:

1. *External phenomena* are perceived in the world model. Their referents are conceived in the material environment of a subject. This class comprises visual, auditory, tactile, olfactory, and kinetic phenomena, the impression of invisible but present agents, inexplicable changes to the body, phenomena concerning audio or visual recordings or the location, structure, or composition of material objects.
2. *Internal phenomena* are perceived in the self model. They include somatic sensations, unusual moods, and feelings, thought insertion, inner voices, and intriguing inner images. As in class (1), the affected subject is convinced that familiar explanations are suspended, and the experiences appear egodystonic.
3. *Coincidence phenomena* refer to experiences of relations between the self model and the world model that are not founded on regular senses or bodily functions, but instead exhibit connections between ordinarily disconnected elements of self and world. Typically, these relations are assumed to be non-causal, often experienced as a salient meaningful link between mental and material events.
4. *Dissociation phenomena* are manifested by disconnections of ordinarily connected elements of self and world. For instance, subjects are not in full control of their bodies, or experience autonomous behavior not deliberately set into action. Sleep paralysis, out-of-body experiences and various forms of automatized behavior are among the most frequent phenomena in this class.

Note that this classification is based on a theoretical approach anchored in the philosophy of mind and offers a systematic perspective on EE. Consequently, our phenomenologically oriented notation is not entirely in line with the historically evolved catalogs of disorder symptoms as in standard psychiatric diagnosis manuals (DSM or ICD). We will show that the empirical material analyzed in this study is in striking agreement with our theoretically founded classification.

### 2.2 Previous results

Since 1996, an empirical database of EE occurrences has been maintained by the counseling department at the Institute for Frontier Areas of Psychology and Mental Health (IGPP) in Freiburg, Germany. As of 2006, that database contains information about 1465 out of the overall 1649 clients with EE, which have been documented in sufficient detail and quality to enable accurate factor analyses. A six-factor solution has proven to yield the most appropriate basis for differentiation and interpretation. Those six factors, ordered by decreasing relative frequency of occurrence, can be phenomenologically described according to the following patterns (Fach, 2011; Belz and Fach, 2012).

*Poltergeist and apparitions (32%)*, within class (1), comprise such phenomena as unexplained movements or changes, disappearing or appearing objects, and sensory impressions without identifiable sources.
*Extrasensory perception (25%)*, within class (3), refers to experiences of coincidences of events without causal connection, but related to one another by some common meaning. They are reported between the inner, mental state of the affected subject and the inner states of others or external physical events.
*Internal presence and influence (23%)*, within class (2), are characterized by somatic phenomena (energy flux, pain) without a medically established explanation, thought insertion, inner voices, or visual impressions that rely exclusively upon internal perception.
*External presence and nightmare (9%)*, within class (4), include phenomena in which an invisible entity-like presence, localized externally, is felt by “atmospheric” or even tactile sensations, and occasionally accompanied by sleep paralysis.
*Meaningful coincidences (6%)*, within class (3), occur between “subjective” inner events and “objective” external events between which no causal relationship is available or seems plausible. Subjects relate them to one another by attributing salient meaning to them.
*Automatism and mediumship (5%)*, within class (4), are EE based on a psychophysical dissociation that differ from external presence. They are often deliberately induced but not controlled, for instance in spontaneous coordinated bodily movements (e.g., automatic writing).

**Figure 1:**
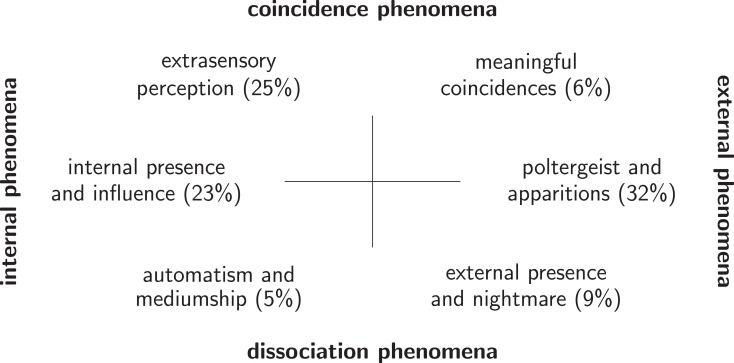
**Empirically obtained patterns (factors) of EE; percentages are relative frequencies of patterns as described in Sec. 2.2**.

It is notable that classes (1) and (2) can be uniquely mapped by the empirical material, whereas classes (3) and (4) are split into two subclasses. Those subclasses can be delineated by a slight dominance of external or internal features in the overly disconnected or overly connected psychophysical relationships that define them. Figure 1 illustrates the six empirically determined patterns and their relative frequencies of occurrence.

## 3 Methods

### 3.1 Empirical material

The empirical material upon which our study was based consists of three subsamples. Two of them refer to clients of the counseling service at IGPP. The remaining subsample, containing a cross section of ordinary Swiss population, was provided by two professional recruiting agencies^2^ commissioned by the Psychiatric University Clinic at Zurich.

The two IGPP subsamples include clients who have reported EE and actively sought advice from counselors at IGPP. Between 1996 and 2006, 1649 client contacts were registered in a documentation system developed at IGPP in the mid 1990s, in accordance with current standards for counseling and psychotherapy. In addition to in-depth reports of the counseling process itself, this system includes a number of modules for recording sociodemographic and clinically relevant data, especially those regarding reported EE (for details, see Belz-Merk et al., 2002; Bauer et al., 2012). These 1649 clients constitute subsample I, from whom the results shown in Sec. 2.2 were obtained.

In addition, a special questionnaire (PAGE-R) for documenting EE was established based on the phenomenology of reported EE. It specifies crucial variables for all types of experienced phenomena^3^. The documentation itself was performed by IGPP personnel, based on reports by the clients. While subsample I consists of the 1649 clients mentioned above, subsample II includes 176 clients who participated in a follow-up survey in 2011.

The Zurich subsample was selected to represent ordinary Swiss population concerning the distribution of gender, age, and education. Recruiters chose a quota sample of 5000 individuals and asked them, via email, to take an online survey based on PAGE-R. The questioning period lasted about 15 min. All 1580 responding individuals, who constitute subsample III (cf. Landolt et al., under review), received a small compensation of CHF 5. The study was approved by the local ethics committee.

In contrast to subsample I, where ratings for clients were expert-assessed by IGPP personnel, the ratings for individuals within subsamples II and III were self-assessed by the respondents. This difference made it possible for us to check whether the different types of assessment might influence the results. Another issue deserving closer scrutiny was that assessments for subsample I usually referred only to EE recently experienced, whereas assessments for subsamples II and III applied to longer-term EE.

### 3.2 Construction of PAGE-R

Based on the analyses of the empirical material from subsample I, IGPP counseling personnel originally had developed an extensive questionnaire (PAGE) for assessing the phenomenology of EE. This questionnaire was later condensed and revised to PAGE-R, which we used to evaluate subsamples II and III.

The EE patterns inquired in PAGE-R reflected the subjective views of the clients about their experiences. As far as accessible, the frequency and time frame of the EE, the clients’ mental state, and the prevailing external circumstances during the EE were assessed as well. It is important to note that the collected data yield an exclusively phenomenological classification scheme, not a system for clinical diagnosis.

Alternative questionnaires for the study of certain kinds of EE are, e.g., the Peters Delusion Inventory (PDI, Peters et al., 1999), the Launay and Slade Hallucination Scale (LSHS, Larøi et al., 2004), and the Diagnostic Interview Schedule for Children (DISC-C, Costello et al., 1982). All these questionnaires study EE more or less from the viewpoint of psychotic symptoms and disorders. By contrast, PAGE-R avoids this preoccupation and focuses on the phenomenology of EE.

The basic structure underlying PAGE-R emerged from the four basic patterns of EE delineated in Section 2.1 (see Fach, 2011; Belz and Fach, 2012). For each pattern, inquiries were made about 8 variables (see Table 1), with five possible ratings of occurrences: 0 (never), 1 (rarely), 2 (sometimes), 3 (often), or 4 (very often).

**Table 1:** **Variables assessed by PAGE-R for the four patterns of EE introduced in Sec. 2.1 according to Fach (2011) and Belz and Fach (2012)**.

External items	Internal items	Coincidence items	Dissociation items
*Exceptional phenomena in the environment*	*Exceptional* *inner perceptions*	*Exceptional* *kinds of knowing*	*Exceptional* *bodily experiences*
Optical phenomena and apparitions	Inner visual images	Present and past events (clairvoyance)	Alterations of the physical body, stigmata
Kinetic changes of physical objects	Strange thoughts, thought insertion	Thoughts and feelings of others (telepathy)	Invisible touch, tactile sensations
Feeling of an invisible presence	Hearing sounds and voices	Meaningful coincidences	Bodily automatisms
Awakening due to external phenomena	Strange emotions and feelings	Anticipation of future events (precognition)	Out-of-body experiences
Noise and acoustic phenomena	Change of character and personality	Places and situations unseen before (déjà vu)	Body-manipulation in sleep
Olfactory phenomena	Somatic sensations	Unveiling hidden facts and a secret order	Bodily paralysis
Thermal phenomena, temperature changes	Contact with entities in dreams	Prophetic dreams of future events	Invisible entities, sexual manipulation
External phenomena with external correspondence	Mental influence of an external source	Correct predictions by oracle techniques	Mediumship and channeling

To minimize confusion about the precise meaning of the variables for participants, items belonging to the four patterns were grouped together with explanatory annotations for each group. Despite early concerns, this did not lead to artificial factor solutions due to sequence effects (see Sec. 4 for further explanation).

In addition, the time of occurrence of EE was rated along a five-graded scale: last 12 months, last 5 years, last 10 years, more than 10 years ago, and before the age of 18. The impact of an experience on an individual was also assessed as 0 (not at all), 1 (a little), 2 (partly), 3 (fairly strong), or 4 (very much).

Finally, PAGE-R contained 12 context variables to specify the circumstances under which the EE occurred: self-induced by own volition, induced by mental techniques, induced by occult practices, drug-induced, occurring spontaneously, occurring in waking state, occurring during contact with healers, occurring in extreme situations, occurring against own volition, valuated as positive and enriching, valuated as negative and burdened, and/or unlikely to recur.

### 3.3 Statistical analyses

The results presented here were obtained through statistical analyses of subsamples II and III. For both, a standard principal component analysis (PCA) was performed over eight variables and four patterns of EE listed in Table 1. The eigenvalue criterion due to Kaiser (eigenvalues exceeding one) was then used to determine the numbers and types of relevant factors.

The assessment of context factors for EE was based on the same type of PCA for the 12 context variables. Finally, sum scores were calculated for the frequency and intensity of EE from subsamples II and III, and also for their corresponding contexts.

Because sum scores are not normally distributed and their variability is fairly large, standard significance tests for differences are suboptimal. Instead, we applied a test procedure in which the responses by all subjects within both subsamples were randomly assigned to two groups with the actual subsample sizes (176 for II versus 1352 for III). If this procedure were applied often enough (e.g., *N* = 10,000), it would produce a robust distribution of sum scores based on a random selection of subgroups.

We then compared the scores from the actual subsamples with this randomized distribution, and computed *p*-values according to the position of the actual scores relative to the distribution. A particular advantage of such a permutation test is that it is based on actual data rather than on more or less plausible model assumptions.

## 4 Results

### 4.1 Phenomenological patterns

Subsample II was based on a follow-up study of 176 clients within subsample I. Restricting the factor analysis to four factors only, we found a classification fully consistent with the theoretical classification presented in Sec. 2.1. The loading plot shown in Table 2 lists the results in detail.

**Table 2:** **Loading values ≥0.40 for the 32 variables in the 4 basic classes of EE for subsample II (IGPP follow-up, *N* = 176)**.

External items	Internal items	Coincidence items	Dissociation items
0.76	0.70	0.80	0.75
Thermal phenomena	Mental influence	Precognition	Manipulation in sleep
0.67	0.64	0.77	0.69
Kinetic phenomena	Somatic sensations	Prophetic dreams	Bodily paralysis
0.64	0.60 (0.41 c)	0.73	0.62 (0.41 e)
Olfactory phenomena	Thought insertion	Telepathy	Tactile sensations
0.52	0.57	0.65	0.57
Acoustic phenomena	Hearing voices	Meaningful coincidences	Bodily alterations
0.51 (0.42 c)	0.55	0.64	0.47 (0.51 i)
External coincidences	Strange feelings	Déjà vu	Sexual manipulation
0.46	0.53	0.62 (0.40 e)	0.44 (0.49 c)
Optical phenomena	Personality changes	Clairvoyance	Out-of-body experiences
0.46 (0.40 i)	– (0.54 c)	0.51	0.44 (0.54 i)
Feeling of a presence	Contact in dreams	Secret order	Automatisms
– (0.52 d)	–	–	– (0.66 i)
Awakening	Visual images	Oracle techniques	Mediumship

*Results were obtained from a PCA that explained 50% of the variance. Insignificant loadings <0.40 were not plotted. Values in brackets show significant loadings for another item class (e, external; i, internal; c, coincidence; d, dissociation)*.

Using the eigenvalue criterion due to Kaiser, we obtained eight rather than four factors for subsample II, in which each of the four basic pattern classes splitted into two subclasses. Interestingly, each set of variables shown in Table 1 gave rise to two factors. This makes it unlikely that the solution was an artifact of lumped variables in PAGE-R^4^.

Table 3 shows the corresponding loading plot for the Swiss online study with 1352 of 1580 participants (participants with no or rare EE were excluded). Although subsample III was comparable in size to subsample I, only four classes again were obtained by the same type of factor analysis. This can be understood as a consequence of the generally lower intensity of EE for the ordinary population subsample versus IGPP clients.

**Table 3:** **Loading values ≥0.40 for the 32 variables in the 4 basic EE classes for subsample III (Swiss online, *N* = 1352)**.

External items	Internal items	Coincidence items	Dissociation items
0.70	0.70	0.76	0.83
Acoustic phenomena	Strange feelings	Precognition	Sexual manipulation
0.64	0.63	0.73	0.82
Thermal phenomena	Personality changes	Telepathy	Mediumship
0.63	0.61	0.68	0.74
Optical phenomena	Thought insertion	Meaningful coincidences	Manipulations in sleep
0.60	0.58	0.68	0.69
Olfactory phenomena	Somatic sensations	Prophetic dreams	Automatisms
0.56	0.56 (0.40 c)	0.68	0.62
Awakening	Visual images	Déjà vu	Tactile sensations
0.53	–(0.53 d)	0.67	0.60
Kinetic phenomena	Hearing voices	Clairvoyance	Bodily paralysis
0.52	–(0.51 c)	0.60	0.52
External coincidences	Contact in dreams	Secret order	Bodily alterations
0.47 (0.46 c)	–(0.47 d)	0.40	0.46
Feeling of a presence	Mental influence	Oracle techniques	Out-of-body experiences

*Results were obtained from a PCA that explained 56% of the variance. Insignificant loadings <0.40 were not plotted. Values in brackets show significant loadings for another item class (c, coincidence; d, dissociation)*.

For both subsamples II and III, all variables loaded on factors consistent with the item groups per PAGE-R. All factors were meaningful and could be interpreted according to the results from subsample I as well as the theoretical expectations described in Section 2.1. This was true even though subsample I referred to IGPP clients seeking advice in an actual situation, while subsamples II and III referred to EE over entire lifetimes.

Because the sample size of the Swiss online study was sufficiently large, we were able to analyze only those participants who had reported EE within the last 12 months (*N* = 349). In this way, we could condense subsample III so that it was more comparable to subsample I. Again, a four-factor solution was obtained by PCA. For each factor, the leading variables were the same as for the full Swiss online subsample, and the differences were marginal in the following variables.

### 4.2 Contexts of occurrences

An additional factor analysis was carried out for subsamples II and III to determine the main classes of contexts in which EE occur. The resulting factors for subsample II are the four columns of Table 4: induced EE, spontaneous EE, conflictual EE, and EE under extreme conditions.

**Table 4:** **Loading values ≥0.40 obtained from a PCA for 12 context variables in PAGE-R**.

	Induced	Spontaneous	Conflictual	Extreme
Mental techniques	0.80 0.65			
Contact with healers	0.76 0.59			
Own volition	0.76			0.48
Occult practices	0.74 0.78			
Spontaneous		0.85 0.76		
Waking states		0.83 0.80		
Positive/enriching	0.48	0.67	−0.79	
Negative/burdened			0.77 0.87	
Against own volition		0.43	0.65 0.68	
Drug-induced			0.58	0.81
Unlikely to recur		−0.45	0.58	
Extreme situations	0.40		0.53	0.71

*Entries on the right within each column refer to subsample II (IGPP follow-up, *N* = 176), those in the left refer to subsample III (Swiss online, *N* = 1352)*.

Analysis of subsample III gave rise to three rather than four factors, with EE under extreme conditions occurring only for subjects within subsample II. This is plausible insofar as that factor being particularly burdensome, such that those concerned individuals tend to seek advice, and thus have an increased likelihood of belonging to subsample II.

It is interesting to note that the valuation of EE as “positive and enriching” did not separate very well between “induced” and “spontaneous” in subsample III, but it did provide negative loading under “conflictual” for subsample II. The opposite, “negative and burdened” clearly loaded under “conflictual” in both subsamples.

Another discrepancy between the two subsamples arose for “drug-induced” and “extreme situations”: II showed high loadings under “extreme conditions,” while both variables loaded at lower levels in subsample III under “conflictual.”

### 4.3 Frequency and intensity of occurrences and contexts

The analysis of the mean values of all 32 EE items yielded that EE occurred less frequently for subjects in the ordinary population of the Swiss online survey (subsample III) than for clients within the IGPP follow-up study (subsample II). The relevant numbers are plotted in Table 5, where the average mean values were 0.99 for II and 0.63 for III.

**Table 5:** **Sum scores for frequencies of EE occurrences in subsample II (IGPP follow-up, *N* = 176) and III (Swiss online, *N* = 1352)**.

		External	Internal	Coincidence	Dissociation	Average
IGPP	Mean	1.04	1.11	1.31	0.49	0.99
Follow-up	SD	0.79	0.82	0.90	0.64	
Swiss	Mean	0.65	0.65	0.90	0.31	0.63
Online	SD	0.65	0.66	0.76	0.51	

*Values are means (and their averages) and standard deviations. Note that standard errors, i.e., standard deviations divided by *N*, would yield even more significant differences between the two subsamples*.

Comparing the individual means in the four patterns, it is apparent that those for IGPP clients exceeded those for Swiss online participants by about 50%. Whereas the most frequent EE in the Swiss online population corresponded to a few clinically inconspicuous phenomena (e.g., feeling of a presence, visual images, or déjà vu), the spectrum of frequent EE was considerably broader for IGPP clients.

Because standard deviations were fairly large for all individual patterns, it was difficult to assess how significant the difference in average mean values was for the two subsamples. A permutation test yielded a distribution of sum scores from randomly selected subgroups that was independent of the partitioning into subsamples II and III but in accordance with their proper sizes. In this way we obtained overall sum scores for many randomly selected subgroups which could then serve as a statistical null hypothesis.

Among the four patterns, scores from the two subsamples for dissociation phenomena deserve extra consideration because the standard deviation greatly exceeded the mean value. For subsample II, this could be explained by the fact that five out of eight dissociation items also loaded on other patterns, often being even higher than for dissociation itself (cf. Table 2). The large variance for subsample III may have had to do with the generally lower intensity of EE in that subsample, which may have made it difficult to render definitive assessments of those items.

**Figure 2:**
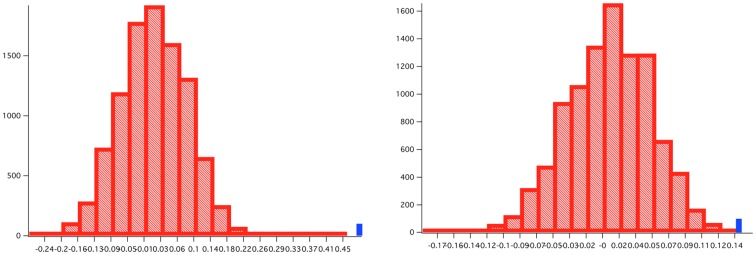
**Distributions of differences in mean values for 10000 randomly permutated subsamples for coincidence phenomena (left) and dissociation phenomena (right)**. The bar in the right wing of the distribution shows the difference of mean values for the actual subsamples (III–II). The probability that this actual difference were compatible with a random permutation of the two subsamples was *p* = 0.0001 in both cases (as well as for internal and external phenomena). Two-sided *t*-tests gave rise to *p* < 10^−9^ for internal, external and coincidence phenomena, and *p* = 0.0002 for dissociation phenomena.

Figure 2 shows the distribution of differences between mean sum scores for those random subgroups as compared to the actually observed differences (III–II) for coincidence phenomena (Figure 2 left) and dissociation phenomena (Figure 2 right). For both EE patterns, the actual difference was located in the wing of the distribution of differences. The *p*-values based on the ratio of the integral of the (smoothly fitted) distribution beyond the actual difference divided by the total integral was *p* = 0.0001 for all four EE patterns (identical values were obtained for medians).

The question of how much the experienced phenomena were still preoccupying participants also yielded different results between the two subsamples. The average for the Swiss online population was 0.85 versus 1.84 for the IGPP follow-up study.

Concerning the contexts of occurrences, the difference in sum scores was remarkable for the valuation of EE. Within subsample III, EE were rated “positive and enriching” with 1.53 (positive) and “negative and burdened” with 0.77 (negative). By contrast, subsample II presented distinct ambivalence in this respect: 2.11 (positive) for “positive and enriching” and 1.70 (negative) for “negative and burdened.”

The higher sum score for negative ratings in the client sample may indicate that their experiences impede on normal functioning – a general criterion for all mental disorders in DSM and ICD. However, because the positive ratings were also enhanced in that sample, this conclusion remains doubtful for the material presented here and should be studied separately.

The other context variables did not differ remarkably. EE occurred predominantly in the waking state, and was mostly spontaneous. Self- or externally induced contexts, such as mental techniques, contacts with healers or persons with esoteric background, or drugs and extreme situations, did not play major roles in either subsample.

## 5 Discussion

As mentioned in the introduction, the empirical material in this study allowed us to compare features of EE in three different directions: (i) clients seeking advice versus subjects within an ordinary population, (ii) recent versus life-long occurrences, and (iii) assessment by experts versus assessment by the experiencing individuals themselves.

Concerning point (i), we compared clients seeking advice, usually prompted by EE that occurred rather recently (subsamples I and II), with individuals in the ordinary population (subsample III). We found that the phenomenology of EE appeared to be organized according to *the same basic structure* of four different EE patterns of phenomena – internal, external, coincidence, and dissociation. Those theoretically derived patterns (Fach, 2011) were empirically identified in all three subsamples, and they were found to be almost identically distributed.

However, we found differences as well. The factor analysis of subsample I provided two significant subpatterns for both coincidence and dissociation phenomena, resulting in six distinguishable factors. The subpatterns became obvious when the overall number of cases was large enough and their intensity and frequency high enough to yield the details needed to differentiate among subpatterns. For instance, the intensity and frequency of EE in subsample III was presumably too low to provide enough details for subpattern identification.

The consistency of basic patterns of EE in all subsamples pointed to an interesting observation that concerns point (ii). Because the material from subsample I contained no longitudinal data about previous EE, its analysis produced information that focused primarily on the actual present states of individuals. By contrast, subjects in subsamples II and III were asked for previous EE and their assessment. This enabled us to infer possible long-time features that might refer to trait-like dispositions. This conjecture was supported by the fact that the basic pattern structure of EE was identical for all subsamples.

The frequency and intensity of EE occurrences over the entire lifespan of individuals differed considerably between subjects in subsamples II and III (I did not contain the necessary data for such a comparison). Subjects in II had frequencies that were about 50% higher than in III, and that difference was significant. The variations in intensity, measured by the question of how strongly previously experienced EE still occupied individuals, were even larger, with II exceeding III by a factor of about 2. These significant differences were highly plausible because subsample II comprised individuals whose EE generated enough concern for them to seek advice, whereas subsample III represented a cross section of ordinary population.

We found that the two subsamples represented two distinguishable non-clinical populations with respect to both frequency and intensity of EE. This significant result supports the hypothesis that the overall spectrum of individuals – from those with severe psychotic, dissociative, or schizotypic symptoms to EE-experiencing clients seeking advice and further to EE in the general population – may in fact be continuous. If so, the naive assumption of a bimodal distribution with a binary distinction between disordered and normal mental functions would be questionable.

The continuum hypothesis proposed by van Os et al. (2000) and others so far has found support mainly for symptoms of severe or less severe mental disorders. Our analysis indicates that this hypothesis may also extend to situations in which EE do hardly or little impede on normal functioning in the lives of individuals. In this sense, the existing material altogether suggests an overall seminormal distribution, where most members of a population are symptom-free, a few are psychotic, and the range between them continuously connects both extremes^5^. We should add, however, that our results for EE cannot be generalized to the full range of mental disorders without further studies based on more comprehensive empirical material.

Remarkably, one of the context issues examined suggests a possible modification of such a seminormal distribution. The question of whether EE are experienced as “positive and enriching” versus “negative and burdened” showed a clear distinction between subsamples II and III. In III, those issues differed by 1.53 (positive) versus 0.77 (negative); II yielded a considerably larger discrepancy of 2.11 (positive) versus 1.70 (negative).

This might indicate that it is appropriate to distinguish positive and negative valuations of EE in a more systematic fashion than a seminormal distribution would allow. If the horizontal axis were extended from positive values only (seminormality) to negative values, the distribution of disorder traits might lead us to a normal distribution capable of distinguishing positively from negatively valuated traits that equally deviate from a symptom-free situation. It remains to be seen up to which amount of disorder this feature is observable.

Point (iii) refers to a comparison between subsamples I and II/III. The results from I were based on a documentation system established and maintained by the IGPP counseling team. Although subjects in II and III responded to a questionnaire developed by that same team, the questions were evaluated by the respondents themselves. Comparing the basic structure of the different EE patterns in those three subsamples, we found no indications for characteristic differences in assessment behavior. This indicates that, within the scope of our study, the variability between self- and expert-assessment did not lead to significantly different results.

## Conflict of Interest Statement

The authors declare that the research was conducted in the absence of any commercial or financial relationships that could be construed as a potential conflict of interest.

## Footnotes

^1^Such deviations are often referred to as “anomalies,” or else “psychic” or “paranormal” experiences. We prefer the notion of a deviation because the theoretical approach taken here *entails* particular basic classes of such deviations. This renders their traditional status as “anomalies” (unrelated to established knowledge) to be at least arguable – if not inappropriate.

^2^Their trade names are panelbiz (www.panelbiz.ch) and respondi (www.respondi.ch).

^3^The acronym PAGE derives from the German “Phänomenologie aussergewöhnlicher Erfahrungen” (phenomenology of exceptional experiences), and the suffix R stands for “revidiert” (revised). The questionnaire is accessible at http://www.igpp.de/questionnaire/index.php?xx=/page&xy=/view-01.

^4^This indicates that sequence effects played no dominant role in our results. We plan to study possible sequence effects in detail and separately in future work.

^5^Another possibility is that psychotic symptoms and EE have so little to do with one another that they should not even be considered as part of the same continuum. At present we have no evidence for such a fairly radical view.
