# Corrigendum: A comparative study of exceptional experiences of clients seeking advice and of subjects in an ordinary population

**DOI:** 10.3389/fpsyg.2015.01414

**Published:** 2015-09-25

**Authors:** W. Fach, H. Atmanspacher, K. Landolt, T. Wyss, W. Rössler

**Affiliations:** ^1^Institute for Frontier Areas of Psychology and Mental HealthFreiburg, Germany; ^2^Collegium HelveticumZurich, Switzerland; ^3^Psychiatric University Clinic ZurichZurich, Switzerland

**Keywords:** continuum hypothesis, exceptional experiences, mental disorders, phenomenological patterns, mental health services

In the article published in Frontiers in Psychology 4, February 2013, article 65, a few entries in Tables 2–4 have been misprinted. The corrected entries are highlighted bold in Tables 2–4 below. The errors in Tables 2, 3 were due to mistaken data import from SPSS into the submitted manuscript. The erroneous positions of entries in Table 4 were due to misprints from the submitted manuscript during copy-editing, and we missed them in the proofs.

**Table 2:** **Loading values ≥0.40 for the 32 variables in the 4 basic classes of EE for subsample II (IGPP follow-up, ***N*** = 176)**.

**External items**	**Internal items**	**Coincidence items**	**Dissociation items**
0.76	0.70	0.80	0.75
Thermal phenomena	Mental influence	Precognition	Manipulation in sleep
0.67	0.64	0.77	0.69
Kinetic phenomena	Somatic sensations	Prophetic dreams	Bodily paralysis
0.64	0.60 (0.41 c)	0.73	0.62 (0.41 e)
Olfactory phenomena	Thought insertion	Telepathy	Tactile sensations
0.52	0.57	0.65	0.57
Acoustic phenomena	Hearing voices	Meaningful coincidences	Bodily alterations
0.51 (0.42 c)	0.55	0.64	0.47 (0.51 i)
External coincidences	Strange feelings	Déjà vu	Sexual manipulation
0.46	0.53	0.62 (0.40 e)	0.44 (0.49 c)
Optical phenomena	Personality changes	Clairvoyance	Out-of-body experiences
0.46 (0.40 i)	**— (0.54 c)**	0.51	0.44 (0.54 i)
Feeling of a presence	**Contact in dreams**	Secret order	Automatisms
— (0.52 d)	—	—	— (0.66 i)
Awakening	Visual images	Oracle techniques	Mediumship

*Results were obtained from a PCA that explained 50% of the variance. Insignificant loadings <0.40 were not plotted. Values in brackets show significant loadings for another item class (e, external; i, internal; c, coincidence; d, dissociation)*.

**Table 3:** **Loading values ≥0.40 for the 32 variables in the 4 basic EE classes for subsample III (Swiss online, ***N*** = 1352)**.

**External items**	**Internal items**	**Coincidence items**	**Dissociation items**
0.70	0.70	**0.76**	0.83
Acoustic phenomena	Strange feelings	**Precognition**	Sexual manipulation
0.64	0.63	0.73	0.82
Thermal phenomena	Personality changes	Telepathy	Mediumship
0.63	0.61	0.68	0.74
Optical phenomena	Thought insertion	Meaningful coincidences	Manipulations in sleep
0.60	0.58	0.68	0.69
Olfactory phenomena	Somatic sensations	Prophetic dreams	Automatisms
0.56	**0.56 (0.40 c)**	0.68	0.62
Awakening	**Visual images**	Déjà vu	Tactile sensations
0.53	**— (0.53 d)**	0.67	0.60
Kinetic phenomena	**Hearing voices**	Clairvoyance	Bodily paralysis
0.52	**— (0.51 c)**	0.60	0.52
External coincidences	**Contact in dreams**	Secret order	Bodily alterations
0.47 (0.46 c)	**— (0.47 d)**	0.40	0.46
Feeling of a presence	**Mental influence**	Oracle techniques	Out-of-body experiences

*Results were obtained from a PCA that explained 56% of the variance. Insignificant loadings <0.40 were not plotted. Values in brackets show significant loadings for another item class (c, coincidence; d, dissociation)*.

**Table 4:** **Loading values ≥0.40 obtained from a PCA for 12 context variables in PAGE-R**.

	**Induced**		**Spontaneous**		**Conflictual**		**Extreme**	
**Mental techniques**	0.80	0.65						
**Contact with healers**	0.76	0.59						
**Own volition**	0.76							0.48
**Occult practices**	0.74	0.78						
**Spontaneous**			0.85	0.76				
**Waking states**			0.83	**0.80**				
**Positive / enriching**	0.48		**0.67**			−0.79		
**Negative / burdened**					0.77	0.87		
**Against own volition**			**0.43**		0.65	0.68		
**Drug-induced**					**0.58**			0.81
**Unlikely to recur**				−0.45	**0.58**			
**Extreme situations**	0.40				**0.53**			0.71

*Entries on the right within each column refer to subsample II (IGPP follow-up, N = 176), those in the left refer to subsample III (Swiss online, N = 1352)*.

The corrected result under internal items in Table 2 shows that “contact in dreams” actually loads under coincidence experiences. This entails a slightly weaker significance of the distinction into basic EE classes for subsample II than in the original publication. The corrected results under internal items in Table 3 move the loadings for the lowest three items to dissociation and coincidence experiences, thus again reducing the significance of the distinctions between classes for subsample III as compared to the original publication. The conclusions of the article remain unchanged.

The construction of the four basic classes from the distinction between self model and world model implies that a perfect distinction between classes cannot be expected anyway. The reason is that the relational classes (coincidence, dissociation) are by definition not independent of the classes (internal, external) between which they are relations. Only in case of full independence could a standard PCA resolve classes perfectly.

Finally, the size of sample III (Swiss online) has been misprinted as *N* = 1532 on p. 4 (right column, third paragraph, line 6) and in Table 5 (caption). The correct sample size is *N* = 1352.

## Conflict of interest statement

The authors declare that the research was conducted in the absence of any commercial or financial relationships that could be construed as a potential conflict of interest.

